# Leisure-time physical activity volume, intensity, and duration from mid- to late-life in U.S. subpopulations by race and sex. The Atherosclerosis Risk In Communities (ARIC) Study

**DOI:** 10.18632/aging.102916

**Published:** 2020-03-13

**Authors:** Dmitry Kats, Kelly R. Evenson, Donglin Zeng, Christy L. Avery, Priya Palta, Stephen B. Kritchevsky, Gerardo Heiss

**Affiliations:** 1 Department of Epidemiology, Gillings School of Global Public Health, University of North Carolina, Chapel Hill, NC 27599, USA; 2 Department of Biostatistics, Gillings School of Global Public Health, University of North Carolina, Chapel Hill, NC 27599, USA; 3 Division of General Medicine, Department of Medicine, Columbia University Medical Center, New York, NY 10032, USA; 4 Sticht Center on Aging, Wake Forest School of Medicine, Wake Forest University, Winston-Salem, NC 27157, USA

**Keywords:** physical activity, exercise, successful aging, healthy aging, retirement

## Abstract

Mitigating age-related disease and disability presents challenges. Physical activity (PA) may be influential for prolonging health and functioning, warranting characterization of its patterns over the life course in population-based data. With the availability of up to three self-reported assessments of past year leisure-time PA (LTPA) over multiple decades in 15,036 participants (26% African American; 55% women; mean baseline age=54; median follow-up=23 years) from the Atherosclerosis Risk in Communities (ARIC) Study sampled from four U.S. communities, race-sex-stratified trajectories of average weekly intensity (metabolic equivalent of task (MET)), duration (hours), and energy expenditure or volume (MET-h) of LTPA were developed from age 45 to 90 using joint models to accommodate expected non-ignorable attrition. Declines in weekly LTPA intensity, duration, and volume from age 70 to 90 were observed in white women (2.9 to 1.2 MET; 2.5 to 0.6 h; 11.1 to 2.6 MET-h), white men (2.5 to 1.0 MET; 3.5 to 1.8 h; 15.5 to 6.4 MET-h), African American women (2.5 to 2.4 MET; 0.8 to 0.1 h; 6.7 to 6.0 MET-h), and African American men (2.3 to 1.4 MET; 1.5 to 0.6 h; 8.0 to 2.3 MET-h). These data reveal population-wide shifts towards less active lifestyles in older adulthood.

## INTRODUCTION

The increase in adults 65 years and older over the coming decades foretells historically high numbers of disease and disability in economically developed societies [1–4]. Consequently, there will be elevated demands for healthcare services and caregivers support [5]. To limit the financial and societal burdens associated with the growth of the elderly population, strategies geared at promoting healthy aging (i.e., preserving health/functioning into older adulthood) are of high priority [6, 7].

Modifiable health behaviors such as physical activity (PA) offer a potentially effective approach. Insufficient PA is one of the leading causes of morbidity and mortality in the United States [8]. Additionally, PA influences disease burden through its cardiometabolic effects on adiposity [9, 10], high blood pressure [11, 12], and diabetes [13]. Epidemiologic studies also suggest that PA may promote the maintenance of physical [14–17] and cognitive function [18] into older age.

While evidence points to the beneficial roles of PA in promoting health and functioning across the adult life course, there has been a scarcity of longitudinal data suitable to track PA from mid-life to older adulthood [19–21] – particularly in U.S. subpopulations. Such limited knowledge of PA behavior constrains understanding of potentially critical age-associated changes in PA among adults transitioning to late-life. With repeated assessment of leisure-time PA (LTPA) in African American and white women and men spanning over multiple decades, data from four U.S. community-based cohorts of the Atherosclerosis Risk in Communities (ARIC) Study [22] provide a valuable opportunity to characterize temporal patterns in PA from mid-life to older adulthood across subpopulations.

Given the high attrition rates common to cohorts with extended follow-up, a joint modeling approach was implemented to test and account for potential informative censoring in missing PA data due to dropout or death. Accommodating informative attrition, longitudinal trajectories of the average weekly LTPA intensity (in metabolic equivalent of task (MET)), duration (in hours), and volume (i.e., intensity x duration = energy in MET-h) [23] are described over the life epoch from age 45 to 90 years among white women, white men, African American women, and African American men.

## RESULTS

Table 1 displays demographic, cardiometabolic, and LTPA characteristics at baseline for the initial total (N = 15,036) cohort sample of 5,816 white women, 5,249 white men, 2,450 African American women, and 1,521 African American men. Baseline variables are also presented according to cohort retention represented by how many times LTPA was recorded in ARIC, including at baseline. Cohort retention, interpreted as the number of visits attended over follow-up (median=23 years), is inversely related to age, female sex, being African American, educational attainment, in addition to characteristics at mid-life including cigarette smoking as well as the presence of hypertension, obesity, and/or diabetes. Considered at mid-life, engagement (yes/no) in LTPA, duration (in h) of average weekly LTPA, and intensity (in MET) of this average weekly LTPA regimen were each associated with cohort retention, as represented by the number of times LTPA was recorded: 1 (just at baseline), 2 (at baseline and at either visit 3 or visit 5), or 3 (at baseline, at visit 3, and at visit 5). African American men experienced the highest rate of attrition due to dropout or death (71%), followed by African American women (64%), white men (62%), and white women (56%).

**Table 1:** Baseline characteristics of ARIC Study participants (aged 45-64 at cohort intake) and according to cohort retention as quantified by the number of non-missing LTPA measurements over follow-up including baseline (i.e., 3, 2, or 1).

		**# of non-missing LTPA measurements**		
	***Baseline***	**3**	**2**	**1**
**Socio-demographic variables** **Age,** years mean (*SD*)	54 (*6*)	52 (*5*)	55 (*6*)	55 (*6*)
**Female** n (%)	8266 (55)	2911 (58)	3911 (54)	1444 (52)
**African American** n (%)	3971 (26)	1005 (20)	1791 (25)	1175 (43)
**<High school education** n (%)	3560 (24)	644 (13)	1827 (25)	1089 (39)
**Not married** n (%)	2849 (20)	866 (18)	1372 (19)	611 (23)
**Behavioral-metabolic factors** **Current cigarette smoking** n (%)	3965 (26)	867 (17)	1991 (27)	1107 (40)
**Obesity (BMI ≥30 kg/m^2^)** n (%)	4161 (28)	1143 (23)	2133 (29)	885 (32)
Hypertension* n (%)	4300 (29)	1402 (28)	1987 (27)	911 (33)
**Diabetes†** n (%)	1511 (10)	193 (4)	813 (11)	505 (18)
**LTPA**				
Average weekly **volume°, MET-h** median (*Q1, Q3*)	6 (*0,16*)	8 (*0,18*)	6 (*0,16*)	1 (*0,13*)
Average weekly **intensity, MET** median (*Q1, Q3*)	3.5 (*1,4.3*)	3.8 (*1,4.5*)	3.5 (*1,4.3*)	2.9 (*1,4.1*)
Average weekly **duration, h** median (*Q1, Q3*)	1.4 (*0,4.0*)	1.8 (*0,4.1*)	1.4 (*0,4.0*)	0.2 (*0,3.2*)
**No LTPA** reported n (%)	5627 (37)	1602 (32)	2684 (37)	1341 (49)

Assessment of LTPA occurred at baseline (1987-1989), visit 3 (1993-1995), and visit 5 (2011-2013).

*Hypertension prevalent if systolic >140 mmHg, diastolic >90 mmHg, or antihypertensive medications reported.

†Diabetes prevalent if fasting glucose ≥126 mg/dL, non-fasting glucose ≥200 mg/dL, meds, or diagnosis reported.

°Note: median(volume) ≠ median(intensity) * median(duration), as the measured LTPA values appearing in these.

data make up a finite set of elements and are thus not closed under scalar multiplication.

### Trajectories of LTPA over the adult life course

**Figure 1:**
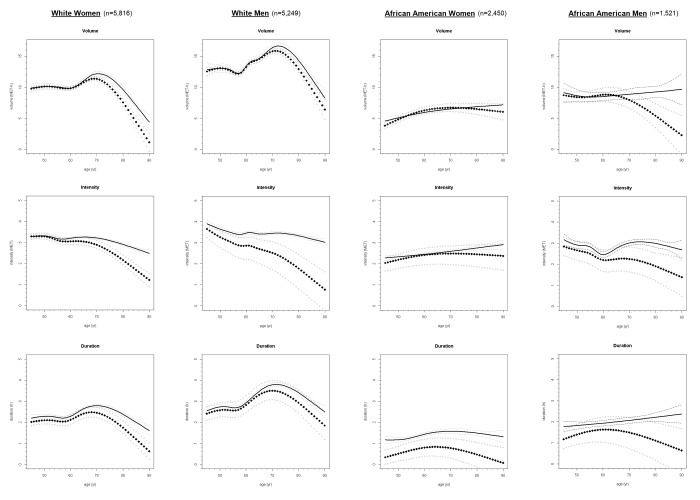
Longitudinal trajectories of average weekly LTPA volume, intensity, and duration from age 45 to 90 in ARIC Study participants (N = 15,036) from joint models* (diamond symbol) and corresponding mixed models† (solid fill), by race and sex. * Trajectories accounting for informative censoring generated through Markov chain Monte-Carlo simulation. † Trajectories fit using only available data (whilst attrition assumed ignorable) via maximum likelihood estimation.

Depicted in Figure 1 are the longitudinal trajectories (along with 95% Confidence Interval (CI) estimates) of average weekly LTPA volume (in MET-h), intensity (in MET), and duration (in h) over the life epoch from age 45 to 90 years as fit using joint models and their corresponding mixed sub-models, by race and sex subgroups. As illustrated by the divergence in trajectories of joint model estimates under those of their originating mixed sub-models over the aging period and confirmed quantitatively through statistically significant (*P* <.05), negative-signed estimates in each joint model output for α, representing the strength of the association between the longitudinal marker (LTPA intensity, duration, volume) and the risk of the event (i.e., right-censoring/missingness), reporting of valid LTPA trajectories necessitated accommodation of an established non-ignorable level of missingness not at random (MNAR) in these data through application of joint modeling. The trajectories of LTPA produced by joint models accounting for informative censoring bias are described over ~5-year age intervals from age 45 to 90 in Table 2. These graphic and tabulated estimates indicate the change in LTPA volume, intensity, and duration across the adult life course among four major U.S. subpopulations.

**Table 2:** Longitudinal estimate with *(lower*, *upper)* 95% confidence bounds for average weekly LTPA volume, intensity, and duration from joint models derived* over ~5-yr intervals from age 45 to 90 in ARIC Study participants (N = 15,036), by race and sex.

	**Age interval**								
	**45-49 yr**	**50-54 yr**	**55-59 yr**	**60-64 yr**	**65-69 yr**	**70-74 yr**	**75-79 yr**	**80-84 yr**	**85-90 yr**
**White Women** (n=5,816)									
**Volume** MET-h	**9.9**	**10.1**	**9.9**	**10.2**	**11.2**	**11.0**	**9.1**	**6.2**	**2.6**
	(*9.6*, *10.3*)	(*9.9*, *10.4*)	(*9.6*, *10.1*)	(*9.9*, *10.5*)	(*10.9*, *11.5*)	(*10.6*, *11.3*)	(*8.6*, *9.6*)	(*5.4*, *7.0*)	(*1.4*, *3.8*)
**Intensity** MET	**3.3**	**3.2**	**3.1**	**3.1**	**3.0**	**2.8**	**2.4**	**2.0**	**1.4**
	(*3.2*, *3.4*)	(*3.1*, *3.4*)	(*2.9*, *3.2*)	(*2.9*, *3.2*)	(*2.8*, *3.2*)	(*2.6*, *3.0*)	(*2.2*, *2.6*)	(*1.7*, *2.2*)	(*1.2*, *1.7*)
**Duration** h	**2.1**	**2.1**	**2.0**	**2.2**	**2.5**	**2.4**	**2.0**	**1.5**	**0.9**
	(*1.9*, *2.2*)	(*1.9*, *2.3*)	(*1.8*, *2.3*)	(*2.0*, *2.5*)	(*2.2*, *2.7*)	(*2.1*, *2.6*)	(*1.8*, *2.3*)	(*1.2*, *1.9*)	(*0.6*, *1.3*)
**White Men** (n=5,249)									
**Volume** MET-h	**12.8**	**12.9**	**12.4**	**14.0**	**15.1**	**15.8**	**14.4**	**11.5**	**7.9**
	(*12.3*, *13.3*)	(*12.6*, *13.3*)	(*12.1*, *12.8*)	(*13.6*, *14.3*)	(*14.7*, *15.5*)	(*15.3*, *16.2*)	(*14.0*, *14.9*)	(*10.9*, *12.3*)	(*6.6*, *9.2*)
**Intensity** MET	**3.5**	**3.1**	**2.9**	**2.8**	**2.6**	**2.4**	**2.0**	**1.6**	**1.0**
	(*3.0*, *3.8*)	(*2.6*, *3.5*)	(*2.3*, *3.3*)	(*2.1*, *3.2*)	(*1.9*, *3.1*)	(*1.6*, *2.9*)	(*1.1*, *2.7*)	(*0.6*, *2.3*)	(*0.0*, *1.8*)
**Duration** h	**2.5**	**2.6**	**2.6**	**3.0**	**3.4**	**3.5**	**3.2**	**2.7**	**2.1**
	(*2.2*, *2.8*)	(*2.3*, *2.9*)	(*2.3*, *3.0*)	(*2.6*, *3.5*)	(*3.0*, *3.9*)	(*3.0*, *4.0*)	(*2.7*, *3.7*)	(*2.2*, *3.3*)	(*1.5*, *2.8*)
**African American Women** (n=2,450)	**45-49** yr	**50-54** yr	**55-59** yr	**60-64** yr	**65-69** yr	**70-74** yr	**75-79** yr	**80-84** yr	**85-90** yr
**Volume** MET-h	**4.2**	**5.2**	**5.9**	**6.4**	**6.7**	**6.7**	**6.6**	**6.4**	**6.1**
	(*3.7*, *4.7*)	(*4.7*, *5.6*)	(*5.4*, *6.4*)	(*5.9*, *7.0*)	(*6.1*, *7.3*)	(*6.0*, *7.4*)	(*5.8*, *7.4*)	(*5.5*, *7.4*)	(*5.0*, *7.3*)
**Intensity** MET	**2.1**	**2.2**	**2.4**	**2.4**	**2.5**	**2.5**	**2.5**	**2.4**	**2.4**
	(*1.7*, *2.4*)	(*1.8*, *2.5*)	(*1.9*, *2.7*)	(*2.0*, *2.8*)	(*2.0*, *2.8*)	(*1.9*, *2.9*)	(*1.9*, *2.9*)	(*1.8*, *2.9*)	(*1.7*, *2.9*)
**Duration** h	**0.4**	**0.6**	**0.7**	**0.8**	**0.8**	**0.7**	**0.6**	**0.4**	**0.2**
	(*0.1*, *0.8*)	(*0.2*, *1.0*)	(*0.3*, *1.1*)	(*0.4*, *1.2*)	(*0.4*, *1.3*)	(*0.2*, *1.2*)	(*0.0*, *1.1*)	(*0.0*, *1.0*)	(*0.0*, *0.9*)
**African American Men** (n=1,521)									
**Volume** MET-h	**8.6**	**8.4**	**8.6**	**8.8**	**8.4**	**7.5**	**6.3**	**4.8**	**3.0**
	(*7.7*, *9.5*)	(*7.6*, *9.3*)	(*7.7*, *9.6*)	(*7.6*, *10.0*)	(*7.1*, *9.7*)	(*6.1*, *9.0*)	(*4.6*, *8.0*)	(*2.6*, *7.0*)	(*0.2*, *5.9*)
**Intensity** MET	**2.8**	**2.6**	**2.4**	**2.2**	**2.3**	**2.2**	**2.0**	**1.8**	**1.5**
	(*2.3*, *3.2*)	(*2.1*, *3.0*)	(*1.9*, *2.8*)	(*1.6*, *2.7*)	(*1.7*, *2.8*)	(*1.6*, *2.8*)	(*1.4*, *2.6*)	(*1.1*, *2.5*)	(*0.7*, *2.4*)
**Duration** h	**1.3**	**1.5**	**1.6**	**1.6**	**1.6**	**1.4**	**1.2**	**1.0**	**0.8**
	(*0.8*, *1.7*)	(*1.0*, *2.0*)	(*1.0*, *2.2*)	(*1.0*, *2.2*)	(*0.8*, *2.2*)	(*0.6*, *2.2*)	(*0.4*, *2.1*)	(*0.1*, *1.9*)	(*0.0*, *1.7*)

* Each ~5-yr interval estimate calculated as weighted average of 1-yr fitted values generated by joint models, with corresponding standard errors as weights.

### Age-related patterns in LTPA

The results from joint models presented in Figure 1 and Table 2 display prominent reductions in the average weekly LTPA intensity (beginning as early as age 45) among white women, white men, and African American men. With declines in intensity accelerating near age 70, intensity fell to ≤1.5 MET when approaching age 90 in these three race-sex subgroups. In contrast, African American women did not exhibit such a pattern of declining intensity levels from mid-life into older adulthood. Instead, we observed a slight increase in intensity in African American women (2.1 to 2.5 MET from age 45 to 70) followed by a decrease – but only to 2.4 MET – by age 90.

Also shown in Figure 1 and Table 2, LTPA duration changed from age 45 years to 90 years in similar trajectories across the subgroups of race and sex. The temporal pattern in LTPA duration represented by these trajectories can be described as an initial rise (2.1 to 2.5 h in white women, 2.5 to 3.4 h in white men, 0.4 to 0.8 h in African American women, and 1.3 to 1.6 h in African American men) from age 45 to 70, followed by a sharp decreases to 0.6 h for white women, 1.8 h for white men, 0.1 h for African American women, and 0.6 h for African American men by age 90. The declines in duration appeared to level off around the same age (70 years) when intensity started to decline in African American women. The fastest rates of decline in intensity were observed among white women, white men, and African American men.

An inflection point in the trajectories of LTPA volume (or energy expenditure) was observed at age 70 across all four race-sex groups. The initial increase in total volume from age 45 approaching age 70 (9.9 to 11.1 MET-h in white women, 12.8 to 15.5 MET-h in white men, or 4.2 to 6.7 MET-h in African American men) shifted at age 70 years. After age 70, LTPA volume reversed its trajectory and by age 90 declined to values of 2.6 MET-h in white women, 6.4 MET-h in white men, 6.0 MET-h in African American women, and 2.3 MET-h in African American men.

### Life-course patterns in LTPA by race and sex

Differences in the average weekly LTPA duration were observed by race and sex subgroups from age 45 to 90 years. Duration of LTPA was highest among white men transitioning into older adulthood, followed by white women, African American men, and African American women. Similar patterns were observed for LTPA intensity and volume until age 75 for African American men, white men, and white women. We did not see declines in intensity of LTPA among African American women, whose intensity levels remained stable across the adult life course. The highest levels of LTPA intensity reported by African American women occurred by age 75 and on. In contrast, white men reported the lowest intensity of LTPA among all race-sex subgroups by age 75.

Despite the declines in intensity, white men expended more energy on LTPA (as captured by volume) compared to the other race-sex subgroups over the entire interval from age 45 to 90 years. Although these energy expenditure estimates in white adults were initially double those of their African American counterparts at mid-life, a decline in total LTPA volume to similarly low levels of 5-7 MET-h occurred by age 80 for all subjects. From age 80 and on, African American women actually showed greater levels of LTPA volume than white women and African American men.

### Role of retirement

To assess the factors that may inform changes in LTPA across the life course, we explored the role of retirement on changes in LTPA [24, 25]. In Supplementary Table 1 and 2, we examined mean differences in the average weekly intensity (in MET) and duration (in h) of LTPA by retirement status across ~5-yr intervals from ages 45-75 years. Differences in intensity and duration were observed across sex, but not race. Retired men engaged in 2.5 (95% CI: 0.2, 4.8) more h of LTPA on average each week compared to men who remained employed at ages 70-75. Average weekly LTPA intensity among retired women was reported to be 1.5 (95% CI: 0.3, 2.7) MET greater than that of non-retired women at ages 70-75.

## DISCUSSION

Trajectories of LTPA volume in U.S. adults were characterized from age 45 to 90 years in a biracial, community-based cohort estimated from repeat measures of PA performed during leisure-time collected over more than two decades. Longitudinal patterns in the average energy expenditure of LTPA (as measured by LTPA volume and across its components of intensity and duration) were estimated across the adult life epoch in four race-sex subpopulations, using joint modeling to account for bias associated with cohort attrition.

African American women and men, on average, showed relatively low levels of energy expenditure (via LTPA) from mid-life to older adulthood. In contrast, white men and women exerted nearly double the energy at baseline as that of their African American counterparts. Among white adults, increases in LTPA expenditure were observed the seventh decade of life, followed by declines in volumes of LTPA that reached levels similar to those of the African American cohort members.

The temporal patterns in PA among free-living adults illustrated by these results are in general agreement with recent reports on PA in European populations. For instance, age was associated with lower PA duration among British adults approaching older adulthood in the Whitehall II cohort [19] and with lower likelihood of PA engagement later in life in a Finnish population-based cohort [20]. The patterns of LTPA levels observed by race and sex in this study are also consistent with reported findings from cross-sectional samples of U.S. adults [26].

Our results relating retirement to LTPA engagement are similar to previous investigations using ARIC data [24, 25]. The differences observed by retirement status provide further indication that retirement from work is associated with greater engagement in PA among older adults. The different patterns observed upon retirement between women and men, specific to the average weekly intensity and duration of LTPA, suggest potential differences in how men and women adopt PA with more available leisure-time. In order to consider policy implications, the results reported here merit replication in other cohorts.

Since the information on PA collected in this study is based on self-report, our data are susceptible to reporting error and possible misclassification bias. The use of device-based measurements of PA, such as by accelerometry, is however challenging for extended follow-up of large cohorts. Given its strong performance against more objective PA measures [27–29, 31], the Baecke questionnaire provides reasonably valid and consistent estimates. While in this study information was only available for sport or exercise activities performed during times of leisure and not for other types of PA, most discretionary PA is in fact performed at leisure-time [29]. Furthermore, a more feasible opportunity for lifestyle intervention is offered during leisure-time in comparison to other domains of activity.

The focus on longitudinal examination of PA over a wide age span is a salient strength of this study, thereby capturing the influence of important life transitions from middle age into older adulthood, including retirement as presented in supplementary analysis. To appropriately evaluate such an extended follow-up period, the use of joint modeling to correct for informative censoring proved to be critical since failing to account for the influence of cohort attrition would have led to inaccurate characterization of LTPA to some degree across all four race-sex groups. Generalizability of the reported estimates is aided by the demographic diversity and population-based nature of the cohort.

Our results identified temporal patterns of LTPA volume, intensity, and duration among African American and white women and men in a population-based cohort from mid-life to older adulthood. Distinct age-related patterns were observed in each LTPA component by race and sex over the course of the adult life epoch. The reported findings can inform the design and testing of lifestyle interventions of the role of PA in the maintenance of health and functioning.

## MATERIALS AND METHODS

### Study population

The community-based ARIC Study cohort consists of 15,792 men and women (of whom predominately reported as white or African American) aged 45-64 years at baseline visit 1 (1987-1989), sampled from four U.S. communities (Forsyth County, NC; Jackson, MS; Minneapolis, MN; and Washington County, MD) [22]. Participants who did not report white or African American, participants reporting African American from Minneapolis or Washington County, in addition to participants with any missing baseline covariate data were excluded (<5% in total), providing a total of 15,036 participants for analysis.

Follow-up examinations to monitor cardiovascular conditions, reassess cardiometabolic factors, and gage social/lifestyle variables took place in 1990-1992 (visit 2), 1993-1995 (visit 3), 1996-1998 (visit 4), and 2011-2013 (visit 5). Institutional review boards at participating sites approved the ARIC Study, and informed consent was obtained from participants at every clinic visit.

### Measurement of PA

Self-reported information related to the type and frequency of PA performed during leisure-time (i.e., LTPA) was collected at baseline, visit 3, and visit 5 in the ARIC Study using a modified version of the Baecke Physical Activity questionnaire [30] administered by trained interviewers through a standardized protocol. The instrument has demonstrated modest correlation to cardio-respiratory fitness (0.5-0.7) and accelerometer-assessed PA (0.6-0.7), a moderate association with alternate self-report assessments (e.g., ~0.5 with PA diary), and consistently high repeatability (e.g., ≥0.6 in men) across various other study populations [28, 29, 31].

Within the modified Baecke, participants were asked whether they exercised or played sports during leisure-time over the past year. Those indicating they did so were requested to list (up to four) activities performed and to estimate the number of hours per week and months of the year they engaged in each activity [31]. Reported activities were assigned their corresponding intensity values (in MET) per the Compendium of Physical Activities. This intensity can be interpreted as the power or work rate (i.e., energy per h) of that activity’s performance relative to what is exerted at rest (or 1 MET, roughly equivalent to 1 kilocalorie per kilogram of body weight per h) [32]. Using this information, continuous estimates of the average weekly duration, intensity, and volume of LTPA were derived as so (for four reported activities):

- Duration is the time (in h) spent on LTPA each week on average, calculated weighting by the proportion of months each activity makes up of the total months of all reported activities:
  h_1_∙ (months_activity 1_ / months_**total**_) + h_2_ ∙ (months_activity 2_ / months_**total**_) +
  h_3_ ∙ (months_activity 3_ / months_**total**_) + h_4_ ∙ (months_activity 4_ / months_**total**_).
- Intensity is the power (in MET) of the average weekly LTPA regimen, calculated weighting by the proportion of the duration of all reported activities represented by each activity:
  MET_1_ ∙ (duration_activity 1_ / duration_total_) + MET_2_ ∙ (duration_activity 2_ / duration_total_)
  + MET_3_ ∙ (duration_activity 3_ / duration_total_) + MET_4_ ∙ (duration_activity 4_ / duration_total_).
- Volume (in MET-h) of LTPA is the arithmetic product of intensity (i.e., power or hourly work rate in MET) and duration (h). As the product of measures of power and time, volume physically represents the total work, or energy expenditure, of the average weekly LTPA.

Reports of no engagement in LTPA are incorporated in analytic samples to provide estimated trajectories applicable to the general population of aging adults, including the considerable proportion of those who do not engage in LTPA. A report of no LTPA is assigned an intensity of 1.0 MET (corresponding to being generally at rest during leisure-time), a duration of 0 h, and accordingly a volume of 0 MET-h.

### Covariates

Sociodemographic variables were self-reported by ARIC participants at baseline including age, sex, white or African American, as well as highest educational attainment (<, =, or > high school). Cardio-metabolic factors were also assessed at baseline. Sitting blood pressures were measured three times following a 5-minute rest. The mean of the last two measurements is applied for classification of prevalent hypertension, indicated by a systolic blood pressure >140 mmHg, a diastolic blood pressure >90 mmHg, or self-reported use of antihypertensive medication. Information on both cigarette and drinking status (current, former, or never) is available from questionnaire response. Baseline anthropometric measurements used in computation of body mass index (BMI) are the quotient of weight (in kg) and squared height (meters^2^). A cut-off of ≥30 kg/m^2^ was employed to index obesity. Prevalent diabetes was identified by a fasting blood glucose ≥126 mg/dL, a non-fasting serum glucose ≥200 mg/dL, self-reported use of hypoglycemic medications (oral or insulin), or self-reported physician diagnosis of diabetes. Occupational status information was self-reported at baseline, at visit 3, and over annual follow-up calls thereafter. For this study, participants who reported working at mid-life are identified as either retired or non-retired (i.e., no longer working versus still employed).

### Statistical analysis

Assuming data are informatively censored (due to cohort attrition), ‘conventional’ strategies for longitudinal analysis of prospective data – such as mixed models, which rely on only the available (i.e., non-missing) measures of the longitudinal outcome [33] – may lead to biased estimation [34]. While application of imputation and weighting methods can provide unbiased estimates when missingness of follow-up data does not depend directly upon the longitudinal variable of interest, these techniques are unable to produce valid (or precise) estimates when missingness in these data occurs under an MNAR mechanism at rates ≥25% [35].

Due to the high rate of dropout common to cohorts with extended follow-up, particularly those that follow large samples of aging adults, it is important to consider the potential influence of informative attrition on the validity of study estimates [34, 35]. Bayesian joint models [36, 37] allow for valid causal inference in the presence of incomplete data, and are particularly well suited for application to the present setting in which death and dropout occur as competing risks. The outcome of missingness or more formally, right-censoring, is quantified as a binary indicator. Bayesian joint modeling proceeds through a Markov Chain Monte Carlo algorithm to generate subsequent increments of simulated information over continuous time.

Age was used as the time scale in this study also as a way to simplify model fitting and provide interpretable LTPA trajectories [38]. Piecewise cubic splines [39] were applied in the fixed effects of mixed sub-models to allow for flexible modeling of marginal (i.e., population-level) trends in the LTPA components over this extended life epoch. Detailed evaluation of likelihood ratio tests, fit statistics, and residual diagnostics, as well as visual inspection guided the placement of spline knots. Splines were not configured into the random effects structure of mixed sub-models, as they did not prove to be necessary and may thus have led to over-specification. A more relaxed random intercepts and slopes structure allowed for adequate model fit of individual deviations in LTPA. Further, a semiparametric Bayesian modeling approach [37] was applied to relax the normality assumption and accommodate the skewness from the aforementioned reports of no LTPA. Baseline values of educational attainment, cigarette smoking, diabetes, obesity, and hypertension were included as covariates in the relative risk sub-models for the missingness process.

For each LTPA measure, assessment of temporal patterns, differences by race and/or sex, as well as informative censoring involved visual and quantitative evaluation. An estimate (noted in the Results as α) was produced in joint models used to assess the presence (test of statistical significance), direction (sign of α), and degree (magnitude of α) of informative censoring. Race-sex specific population-based trajectories of LTPA volume, intensity, and duration were illustrated by combining and superimposing each set of 1-yr estimates (and 95% CI bounds) from age 45 to 90 generated by joint models and corresponding mixed sub-models. Estimates from joint models were quantified over ~5-yr intervals from age 45 to 90. All statistical procedures were performed in R v3.4.0.

## Supplementary Material

Supplementary Tables
