# Supplementary material for: Leisure-time physical activity volume, intensity, and duration from mid- to late-life in U.S. subpopulations by race and sex. The Atherosclerosis Risk In Communities (ARIC) Study
Source: Aging (Albany NY). 2020 Mar 13;12(5):4592–602. doi: 10.18632/aging.102916 (PMC7093185; doi:10.18632/aging.102916)
Supplement: Supplementary Tables [file aging-12-102916-s001..pdf]

SUPPLEMENTARY TABLES

Supplementary Table 1. Estimated mean differences with (lower, upper) 95% confidence bounds in the average weekly duration of LTPA (in h) by retirement status for women and men across ~5-yr age groups.

	**Age group**					
	**45-49 yr**<br>295 retired;<br>1251 not retired	**50-54 yr**<br>218 retired;<br>714 not retired	**55-59 yr**<br>184 retired;<br>169 not retired	**60-64 yr**<br>138 retired;<br>80 not retired	**65-69 yr**<br>109 retired;<br>42 not retired	**70-75 yr**<br>47 retired;<br>17 not retired
Women (n=4371)	0.3 (-0.1, 0.8)	-0.6 (-9.9, 0.0)	0.1 (-0.7, 0.8)	-0.5 (-1.4, 0.5)	0.1 (-1.4, 1.5)	1.5 (-1.0, 3.9)
Men (n=3224)	0.0 (-0.1, 0.5)	0.4 (-0.3, 1.1)	0.4 (-1.3, 1.1)	0.6 (-2.1, 0.9)	1.1 (-0.5, 2.7)	**2.5 (0.2, 4.8)***

* *p<.05.*

Supplementary Table 2. Estimated mean differences with (lower, upper) 95% confidence bounds in the average weekly intensity of LTPA (in MET) by retirement status for women and men across ~5-yr age groups.

	**Age group**					
	**45-49 yr**<br>295 retired;<br>1251 not retired	**50-54 yr**<br>218 retired;<br>714 not retired	**55-59 yr**<br>184 retired;<br>169 not retired	**60-64 yr**<br>138 retired;<br>80 not retired	**65-69 yr**<br>109 retired;<br>42 not retired	**70-75 yr**<br>47 retired;<br>17 not retired
Women (n=4371)	0.3 (0.0, 0.6)	-0.3 (-0.6, 0.1)	0.1 (-0.5, 0.6)	-0.2 (-0.9, 0.4)	**0.4 (-0.1, 0.9)**†	**1.5 (0.3, 2.7)***
Men (n=3224)	0.6 (-0.1, 1.3)	0.4 (-0.1, 0.9)	0.3 (-0.3, 0.9)	-0.2 (-1.1, 0.7)	0.7 (-0.2, 1.6)	0.7 (-0.7, 2.1)

* *p<.05.* † *p<.10.*
